# Properties of Red-Mud-Modified Basic Magnesium Sulfate Cement

**DOI:** 10.3390/ma17164085

**Published:** 2024-08-17

**Authors:** Yanrong Wang, Zhilei Zhen

**Affiliations:** College of Urban and Rural Construction, Shanxi Agricultural University, Mingxian South Road 1st, Taigu, Jinzhong 030800, China; wyr@sxau.edu.cn

**Keywords:** hydration products, mechanical properties, pore structure, microstructure

## Abstract

This study aimed to decipher the influence of red mud on the mechanical properties, pore structure, and microstructure of basic magnesium sulfate cements (BMSCs). The results showed that BMSC prepared with an appropriate addition of red mud exhibited improved mechanical properties and yielded the highest compressive strength of 94.54 MPa after curing for 28 days. Adding red mud reduced the total porosity and optimized the pore structure of BMSC. The microstructure and hydration products of the specimens were examined using X-ray diffraction, scanning electron microscopy, and energy-dispersive spectroscopy. The results illustrate that the addition of 50% red mud did not affect the amount of the main strength phase 5-1-7 produced in BMSC. It could also reduce the residual amount of MgO and the generation of Mg(OH)_2_. The red mud and the M-S-H gel generated by the reaction between active SiO_2_ and α-MgO in the red mud together filled the pore structure of BMSC, making its microstructure denser and higher-strength. This study aims to improve the comprehensive use of red mud, and the results show that red mud can improve the mechanical properties of BMSCs, protecting the environment and simultaneously reducing BMSC production costs to create good economic benefits.

## 1. Introduction

Red mud (RM) is the main solid waste discharged by the aluminum industry [1]. It has been shown that the weight of RM discharged is 1.0 to 1.8 times that of the aluminum oxide produced [2,3]. China is the largest alumina producer in the world, and according to data from the Chinese National Bureau of Statistics, the alumina production in China reached 81.862 million tons in 2022. Considering the generation of RM in alumina production, the RM discharged in 2022 was approximately 120 million tons. The cumulative storage now exceeds 1 billion tons.

RM has strong alkalinity (pH = 10–12.5), can be slightly radioactive [4], and its large-scale storage not only wastes land resources but also poses environmental and safety risks [5,6]. However, RM is widely used for the extraction of valuable metals [7,8], while also being a fertilizer, soil modifier [9,10,11], adsorbent [12,13,14,15], and building material [16,17,18,19,20]. Among these, the production of building material stands as one of the most effective ways to improve the comprehensive utilization of RM [21,22]. Cement can block the radioactivity of RM [23]. The cementing and encapsulation of cement form a dense curing body that adsorbs and wraps the radionuclides in RM, and the curing body can block the penetration of rays, thus realizing the shielding effect on radioactive rays. Hence, it has been widely utilized to prepare building concrete without affecting its performance. Moreover, it also improved the sulfate resistance of concrete and displayed an excellent weight loss effect in the freeze–thaw cycle [24]. The optimal RM-to-cement weight replacement rate was 10%, and more RM leads to a decreased compressive and tensile strength of concrete [25]. The properties of magnesium phosphate cement (MPC) modified by RM were also studied, evidencing an improvement in the mechanical properties and water resistance of the MPC through the addition of a proper amount of RM [26]. Concurrently, RM can fill the cement micropores, thereby increasing its density and forming new cementitious hydrates. A common Portland cement mixed with 20% RM was developed through cement hydration and geopolymer reactions, and its hardening physical properties were better than those of the 32.5R cement [27]. Since RM has cementitious activity, using it as a cement admixture can produce cement with excellent performance [28]. However, RM has both high Na_2_O content and alkalinity, which are not conducive to the formation of calcium silicate hydrate gels, therefore limiting the application of RM in concrete and Portland cements.

Basic magnesium sulfate cement (BMSC) is a main hydration product formed by mixing an appropriate proportion of α-MgO, MgSO_4_·7H_2_O, and a suitable admixture. It is a new cementitious insoluble material with a dense structure composed of a BMS whisker (5Mg(OH)_2_·MgSO_4_·7H_2_O, 5-1-7) [29,30]. BMSC has the characteristics of high and early strengths and water, corrosion, and carbonation resistances, and its physicochemical, mechanical, and reinforcement protection properties are similar to those of Portland cement [31,32]. Furthermore, the light-burned magnesia applied to produce BMSC is calcined at a low temperature, enabling BMSC to meet the building energy saving and environmental requirements. BMSC is therefore anticipated to become a high-performance environmentally friendly cement and presents considerable future development potential [33,34].

Although the performance of BMSC is excellent, owing to the existence of many pores between its hydration products, strength loss after the long-term immersion in water is obvious if no other mineral or functional admixtures are added [34]. The incorporation of fly ash to BMSC improved its compressive strength and volume stability [35], while that of sulfur-fixing ash also enhanced its compressive strength, thereby promoting BMSC comprehensive utilization [36]. Similar to fly and sulfur ashes, RM is a new supplementary cementitious material [25]. Recently, research has reported on the preparation of concrete and Portland cement from RM [37,38,39], but only few elucidated the effect of RM on BMSC. Adding RM to BMSC not only reduces the production cost of cement, but also improves the RM utilization rate. RM’s color results from its high iron oxide (Fe_2_O_3_) content; therefore, it can be used to prepare colored cement. The addition of RM (50%) to magnesium oxychloride and magnesium oxysulfate cements resulted in a significant increase in both water resistance and compressive strength [40]. Therefore, RM exhibits considerable potential to be used as a modifier for magnesium cements, which may affect the mechanical properties and microstructure of BMSC.

In order to enhance the comprehensive utilization of RM and simultaneously reduce the production cost of BMSC to generate positive economic outcomes, red-mud-based magnesium sulfate cements (RM-BMSCs) were prepared in this study, and the influences of different RM contents on the mechanical properties of BMSC were analyzed. The microstructure and hydration products of the specimens were analyzed using X-ray diffraction (XRD), scanning electron microscopy (SEM), and energy-dispersive spectroscopy (EDS). The underlying mechanism enabling the improvement in BMSC properties through RM incorporation was clarified, thereby providing a scientific basis for the research and application of RM-BMSC in the construction industry.

## 2. Materials and Methods

### 2.1. Materials

RM-BMSC was prepared using light-burned MgO, RM, MgSO_4_·7H_2_O solution, and citric acid (C_6_H_8_O_7_) in different proportions. Light-burned MgO was supplied from Liaoning Yingkou Huiteng Refractory Co., Ltd. (Liaoning, China), and an active magnesia content of 60% was determined using the water method. Bayer RM, purchased from Shandong Aluminum Co., Ltd. (Zibo, China), was oven-dried at 105 °C for 24 h and ground in a ball mill, with a particle size of less than 200 mesh after sieving. Industrial magnesium sulfate (MgSO_4_·7H_2_O) was obtained from Shanghai Minhang Shenlong Light Chemical Co., Ltd. (Shanghai, China), and was further diluted to produce a 25% magnesium sulfate solution. Citric acid (C_6_H_8_O_7_) was supplied by Tianjin Beichen Fangzheng Reagent Co., Ltd. (Tianjin, China).

### 2.2. Specimen Preparation

The molar ratio of n(α-MgO):n(MgSO_4_·7H_2_O) was 8:1 and the concentration of C_6_H_8_O_7_ was 1% (α-MgO mass record). In this study, the influence of RM addition on the performance of BMSC was analyzed using RM-to-BMSC mass ratios of 0, 0.1, 0.2, 0.3, 0.4, and 0.5 (Table 1).

The samples were divided into five groups and mixed in a planetary cement sand mixer (NJ-160, Maifang Instruments and Equipment Co., Ltd., Wuxi, China) in the laboratory, prepared according to the following steps: (1) RM, light-burned MgO, and citric acid were dry-mixed at a low speed for 90 s. (2) A 25% MgSO_4_ solution was added, mixed at low speed for 30 s, and then left standing for 90 s. Subsequently, the mixture was stirred at high speed for 60 s. (3) The slurry was injected into a 40 × 40 × 160 mm PVC mold and vibrated for 60 s on a cement vibration table. Excess mud was scraped off with a scraper to make the surface smooth. After 24 h of natural curing, the mold was released and cured at a temperature of 20 ± 3 °C and a relative humidity of 40 ± 5% until the testing age was reached.

### 2.3. Strength Measuerment

According to the Chinese national standard (GB/T17671-2021) ISO test method [41], strength tests were conducted on cement specimens measuring 40 × 40 × 160 mm with curing ages of 1, 3, 14, 21, and 28 days. Flexural tests were conducted using a universal testing machine (WE-300B, Jinan Zhongluchang Testing Machine Manufacturing Co., Ltd., Jinan, China) at a loading rate of 50 N/s. The flexural strength of the test specimens was determined by the average value of three test specimens. The flexural strength was calculated using the following formula:

$$R_{f}=\frac{1.5F_{f}L}{b^{2}}$$

where R_f_ is the flexural strength (MPa), F_f_ is the specimen failure load (N), L is the span (mm), and b is the specimen cross-section length (mm).

After the flexural strength test, the specimens underwent a compressive strength test at a loading rate of 2400 N/s. The average values of all tests were considered the final compressive strength. The compressive strength was calculated as follows:

$$R_{c}=\frac{F_{c}}{A}$$

where R_c_ is the compressive strength (MPa), F_c_ is the ultimate load (N), and A is the compression area (mm^2^).

### 2.4. Microstructure Analysis and Mineralogical Characterization

Following the strength test, the collected broken specimens were immersed in anhydrous alcohol for 7 days to terminate the hydration process. The pore distribution of the specimens was determined using an automatic mercury porosimeter (Micromeritics, AutoPoreIV 9500, Norcross, GA, USA) and Brunauer–Emmett–Teller (BET) nitrogen adsorption branch model (Quadrasorb Anton Parr). The sample should be pulverized into a fine powder and the hydration products of the specimen were analyzed using X-ray diffractometry (XRD) (XD8 Advance, Bruker, Karlsruhe, Germany) with Cu Kα radiation at 60 kV and 80 mA and a scanning ratio of 0.02°/s. The morphology of the specimen was observed using a scanning electron microscope (SEM) (Gemini 300, ZEISS, Oberkochen, Germany). The resolution of SEM was 0.6 nm and the acceleration voltage was 15 kV. Then, the thin slice samples were sprayed with gold and observed by SEM. The elemental composition of the specimen was characterized by an EDS spectrometer (SU8100, Hitachi, Tokyo, Japan). Sample testing was completed at the Hongrui Research Service Center (www.jdhr-test.com; accessed on 7 January 2024).

## 3. Results and Analysis

### 3.1. Properties of the Raw Materials

**Figure 1:**
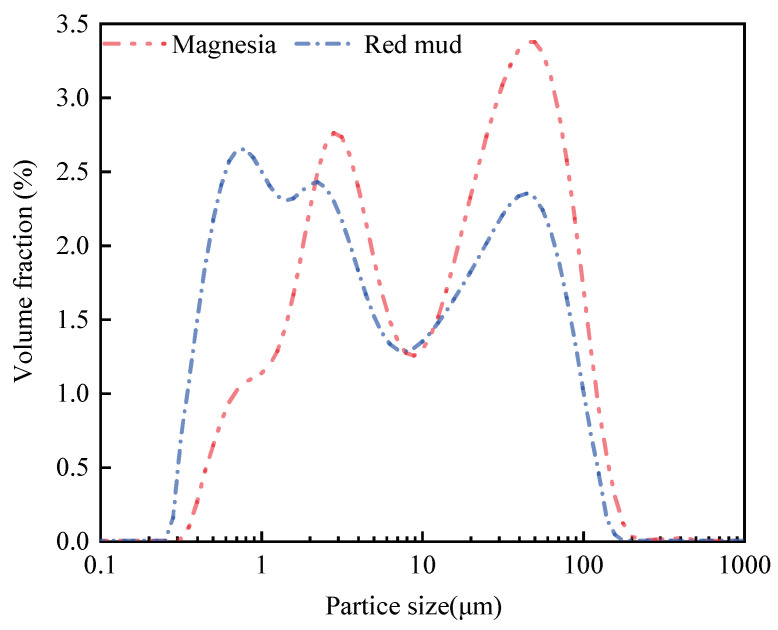
Particle size distribution of magnesia and RM.

**Figure 2:**
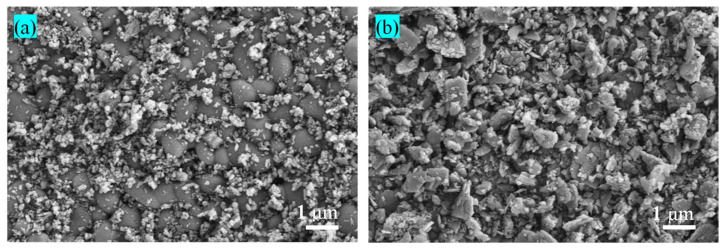
SEM images of (**a**) magnesia and (**b**) RM raw materials.

**Figure 3:**
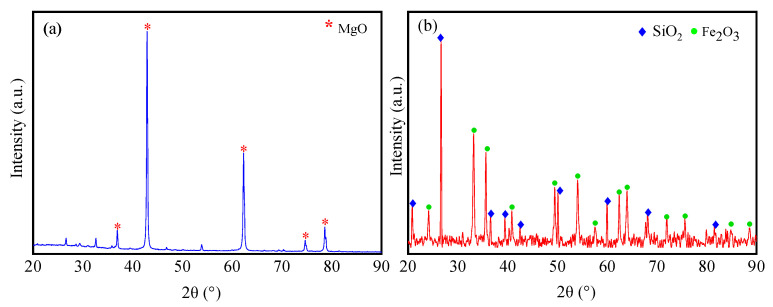
XRD patterns of (**a**) magnesia and (**b**) RM raw materials.

The particle size distribution of magnesia is larger and less uniform than that of RM (Figure 1). The SEM images of magnesia and RM evidence that most of the particles in magnesia are spherical with a regular and uniform distribution (Figure 2a), whereas the particles in RM are small flakes and irregular polygons (Figure 2b). Compared with magnesium oxide, RM has smaller particles and a denser distribution. XRD analysis of magnesia and RM shows that the main phase of lightly burned magnesia is magnesia while silicon dioxide (SiO_2_) and Fe_2_O_3_ are detected in the diffractogram of RM (Figure 3). X-ray fluorescence (XRF) analysis shows that the Fe_2_O_3_ and SiO_2_ contents in RM account for 93.06% of the total oxide, while the MgO content in light-burned magnesia is 93.8% (Table 2).

### 3.2. Mechanical Properties

**Figure 4:**
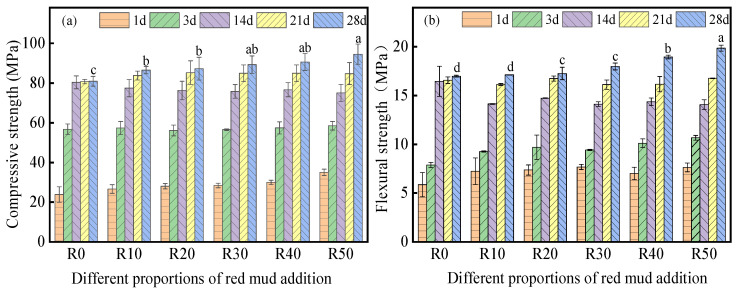
(**a**) Compressive and (**b**) flexural strengths of BMSC with various red mud contents. The same letters indicate no significant difference.

The compressive and flexural strengths of BMSC with different RM additions are shown in Figure 4. Adding RM improved the compressive and flexural strengths of BMSC, and the strengths were highest for sample R50 after curing for 28 days, reaching 94.54 and 19.84 MPa, which outperformed the benchmark (R0) by 17.6% and 16.84% respectively. As the curing time increased, the mechanical properties of the BMSC specimens progressively increased. As the reaction progressed, more hydration products accumulated, increasing the density of the internal structure and improving mechanical strengths. It is observed that the strength of BMSC with different amounts of RM increased rapidly in the first 14 days, but then slowed from 14 to 28 days. Initially, the content of α-MgO and the concentration of MgSO_4_ were higher, and the hydration reaction of the BMSC was faster; thus, the strength increased rapidly. As the reaction progressed, the α-MgO content and MgSO_4_ concentration decreased, and the hydration reaction weakened or even stopped, resulting in a slower increase in the strength [42].

The BMSC compressive strengths of different RM dosages did not differ much at 1, 3, and 14 days. After curing for 14 days, the compressive strengths of R0, R10, R20, R30, R40, and R50 were 80.36, 77.44, 76.26, 75.80, 76.65, and 75.04 MPa, respectively (Figure 4a). This indicates that the RM had almost no effect on the compressive strength of BMSC in the first 14 days. After 14 days of curing, the compressive strength of BMSC gradually increased with increasing RM content. After curing for 28 days, the compressive strength of R0 and R50 had a significant difference; the difference between R10, R20, R30, and R40 was not significant; and the compressive strength of R0 and R50 was significantly different from that of R10, R20, R30, and R40. The compressive strengths of R0, R10, R20, R30, R40, and R50 were 80.93, 86.51, 87.26, 89.28, 90.60, and 94.54 MPa, respectively. Compared with the R0 specimen, the compressive strength of the BSMC mixed with 50% RM increased by 17.6% after 28 days of curing. These results evidence that the RM had little effect on the early strength of BMSC but increased the 28 days compressive strength. This is because red mud only slows down the hydration reaction of BMSC but cannot completely block the reaction between MgO and BMSC. Although the hydration reaction is slow in the early stage after the addition of RM, there is less 5-1-7 phase, but the micro-aggregate effect of RM makes up for the early strength. With the solidification of the test block, the BMSC hydration reaction gradually slows down or even stops at 21 days, and fine RM particles continue to act in the BMSC, making the BMSC system denser and stronger [43]. Concurrently, with the increase in RM addition, the water–cement ratio of the BMSC decreases, thereby enhancing its strength.

The variation in the BMSC flexural strength with different RM contents is like that of the compressive strength (Figure 4b) as it increases rapidly before 14 days of curing. After 14 days, the flexural strength of R0 does not increase, while that of BMSC with RM contents of 10–50% still slowly increases. After curing for 28 days, the difference between the flexural strength of R10 and R20 is not significant, similar to R30 and R40. The flexural strength of R50 is significantly different from those of R0, R10, R20, R30, and R40. The flexural strength of R50 is highest at 19.84 MPa.

### 3.3. Effect of RM on the Pore Structure of BMSC

**Figure 5:**
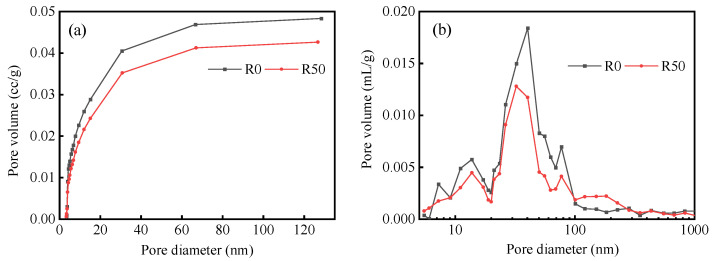
(**a**) Mesoporous and (**b**) macroporous pore diameter distribution of BMSC specimens cured for 28 days.

Figure 5a,b show the mesoporous (2–50 nm) and macroporous (>50 nm) pore distributions of the specimens determined by BET nitrogen adsorption and using an automatic mercury porosimeter, respectively. As compared with R0, the macroporous and mesoporous volumes of the R50 specimens are lower. The porosity of R0 (22.09%) is higher than that of R50 (18.07%); the total pore volume, average pore size, and total pore area of R0 are all greater than those of R50; and its apparent density is lower than that of R50 (Table 3).

These results demonstrate that RM creates micro-aggregates in the BSMC, which effectively fills the internal pores of the specimens, thereby reducing the porosity. Smaller RM particles can increase the density of the BSMC structure [43]. The lower the porosity, the finer the pore structure, and the higher the material strength [44], which is in accordance with the observed results for the mechanical properties of the BMSC specimens. Furthermore, the dense pore structure can regulate the diffusion and leaching of nuclide ions, thereby achieving the shielding effects against radiation. This results in RM-BMSCs exhibiting a lower radioactivity than RM.

## 4. Discussions

**Figure 6:**
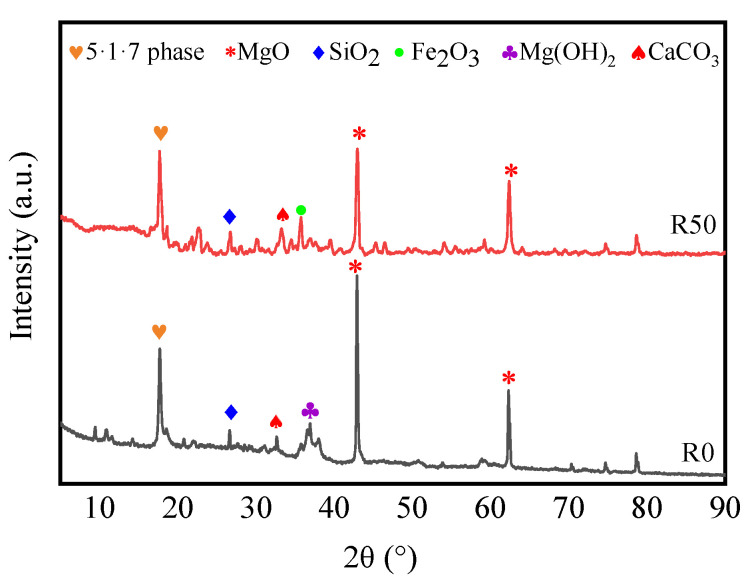
XRD patterns of R0 and R50 cured for 28 days.

Specimens R0 and R50 cured for 28 days were analyzed using XRD to explore the effect of RM on the structural characteristics of the BMSC. As presented in Figure 6, the major hydration products of R0 and R50 only slightly changed after being cured for 28 days. For both specimens, the main phase present is the 5-1-7 phase, but there is also a large amount of unreacted MgO. There is no significant difference in the 5-1-7 phase diffraction peak-to-peak intensity between R0 and R50. However, the MgO diffraction peak-to-peak intensity in R50 is significantly weaker than that in R0, and no Mg(OH)_2_ diffraction peak is found in R50. The microstructure and the ratio of the 5-1-7 phase to Mg(OH)_2_ both strongly affect the compressive strength of magnesium sulfide cement [31]. In our experiment, after 50% RM was added to BMSC, the intensity of the 5-1-7 phase diffraction peak remained the same and the Mg(OH)_2_ diffraction peak disappeared; thus, the 5-1-7 phase/Mg(OH)_2_ ratio remained high and thus the mechanical properties of R50 were better than R0.

The absence of Mg(OH)_2_ and the reduced MgO content indicate that the RM may have reacted with these compounds. The reaction between active SiO_2_ and active MgO forms magnesium silicate hydrate (M-S-H) gelling, which can improve the mechanical properties of BMSC [36]. These cementitious materials and the residual RM can fill microcracks and pores to form a denser structure. Simultaneously, cementitious materials can enhance the bonding properties among particles and improve the strength of the material [45].

**Figure 7:**
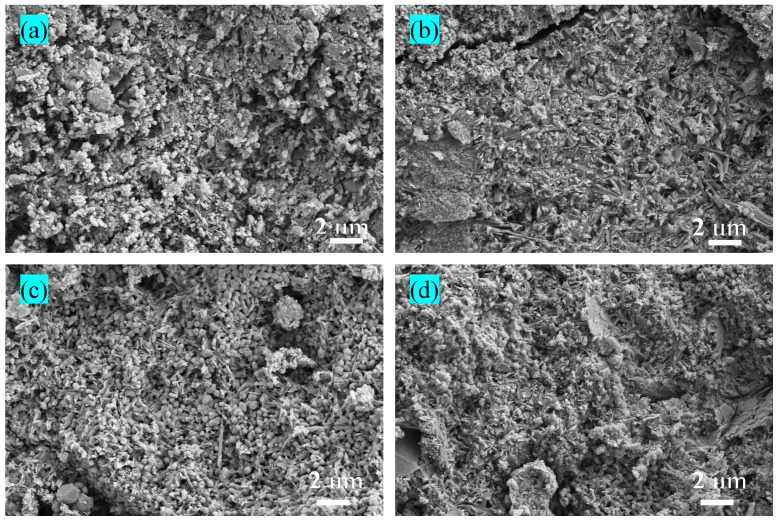
SEM images of BMSC specimen R0 cured for (**a**) 3 days and (**b**) 28 days, and of specimen R50 cured for (**c**) 3 days and (**d**) 28 days.

SEM images of R0 and R50 specimens cured for 3 and 28 days, respectively, show a reduction in the amount of microcracks and micropores and an increasing number of needle-like particles with increasing curing time (Figure 7). This is the result of both the structure of the hydration products that becomes denser and their shape that becomes more regular with increasing curing time. RM can fill the microcracks between the particles of the cement material, therefore densifying the structure and forming new hydrates [46]. After curing for 28 days, the hydrate distribution in R50 is more uniform and the structural density is higher as compared with R0, indicating that RM has created micro-aggregates in BMSC, which can effectively fill the internal voids and densify the cement microstructure.

According to the SEM images of BMSC cured for 3 days, it is observed that R50 contained less needle-like 5-1-7 phase material and more spherical MgO particles as compared with R0 (Figure 7a), indicating that the α-MgO particles were wrapped in fine RM particles, which slowed the hydration reaction of α-MgO. The surface of R50 is smoother, its structure is more compact, and the pores are increasingly smaller with increasing curing time, indicating that the RM particles are embedded in the larger MgO particles, which greatly improves the compactness. Accordingly, R0 and R50 have similar compressive strengths and R0 has a higher flexural strength than R50 after curing for 3 days. This corresponds to the findings of Section 3.2.

In the SEM image of R50 cured for 28 days (Figure 7d), more acicular 5-1-7 phase particles are observed, and the amount of unreacted MgO spherical particles decreases as compared with the specimen cured for 3 d. RM contains a large amount of active SiO_2_ that reacts with α-MgO at room temperature to form M-S-H gel [36]. These substances combine closely with the 5-1-7 phase to fill the internal pores, which makes the cement matrix compact and improves the overall strength of BMSC. Simultaneously, the unreacted red mud particles, which are well embedded in the system, play a filling role. In addition, a suitable addition of mineral admixture can reduce the hydration heat of the cement [47], thereby reducing the number of fine cracks caused by the exothermic process and improving the compressive strength.

Representative R0 and R50 specimens were analyzed using SEM-EDS in a selected region of the backscattered electron image (BES). The BES shows that the BMSC specimen mixed with 50% RM is denser and has no cracks on its surface, evidencing that the addition of RM optimizes the pore distribution and densifies the microstructure.

**Figure 8:**
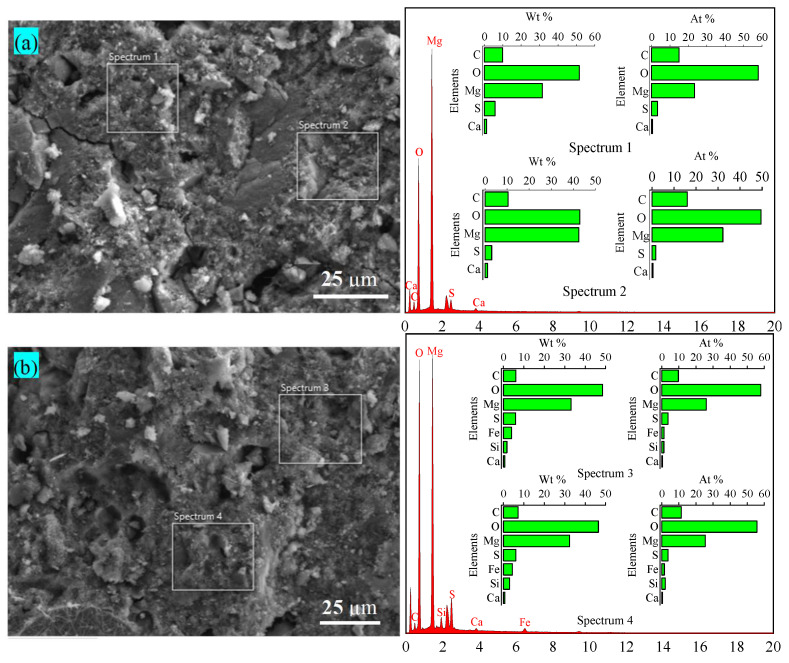
BES images and EDS elemental analysis of the BMSC specimen (**a**) R0 and (**b**) R50 cured for 28 days.

In Spectrum 1 of the EDS elemental analysis of specimen R0 (Figure 8a), the molar ratios of Mg:S:O:C:Ca are 7.06:1:17.58:4.52:0.15, indicating the generation of the 5-1-7 phase and MgCO_3_. In Spectrum 2, the molar ratios of Mg:S:O:C:Ca are 18.94:1:29.12:0.94:0.29, which could be attributed to the presence of the 5-1-7 phase, Mg(OH)_2_, and unreacted residual MgO.

In Spectrum 3 of the EDS elemental analysis of specimen R50 (Figure 8b), the molar ratios of Mg:S:O:C:Fe:Si:Ca are 7.22:16.08:2.69:0.36:0.33:0.08, which suggest the presence of a 5-1-7 phase, MgO, Fe_2_O_3_, and M-S-H gel material, indicating that RM, the cementitious material, and the 5-1-7 phase are embedded together. The molar ratios of Mg, S, O, C, Fe, Si, and Ca in Spectrum 4 are very similar to those in Spectrum 3, with values of 7.08, 15.47, 0.42, 0.52, 0.56, and 0.08, respectively. With the introduction of elements such as Si and Fe, some gelatinous substances attached to the surface of the 5-1-7 phase. Hence, the large amount of active SiO_2_ in the RM reacted with the active MgO to form M-S-H gel [36]. These substances combined closely with the 5-1-7 phase, filling the internal pores, making the internal structure denser, reducing the amount of remaining MgO and Mg(OH)_2_, and improving the overall strength of BMSC. It has been reported that the cement strength increases as porosity decreases, and that the addition of external materials densifies the microstructure and also improves the strength [43]. The microstructure observed in the BSE images evidenced that the density of BMSC mixed with 50% red mud was higher, and there were fewer microcracks as compared to the benchmark specimen, indicating that red mud and the newly generated cementitious materials can fill the internal pores of BMSC, enhance the microstructure, and increase the strength. This is consistent with the results of the mechanical properties in Section 3.1.

It has been reported that the optimal substitution rate for concrete prepared using RM instead of Portland cement is 15%, and that adding more than 15% is detrimental to its mechanical properties [48]. When RM replaces light-burned magnesia in the production of MPC, the best substitution rate is 20%, since adding more than 20% negatively affects the water resistance and mechanical properties of the cement [25]. While the reported optimal content of RM in Portland concrete systems and magnesium phosphate cement is 15% and 20%, respectively, it can reach 50% in BMSC, indicating that the integration of RM in magnesium cement products has significantly greater potential to become an effective resource utilization method for RM.

## 5. Conclusions

(1) The addition of red mud to BMSC had no effect on the early strength but can improve the mechanical properties of BMSC after 28 days of curing. The compressive and flexural strengths of BMSC containing 50% RM reached 94.54 and 19.84 MPa after 28 days of curing, which were highest among all groups.

(2) The addition of red mud can reduce the porosity of BMSC, and the porosity of R0 and R50 is 22.09% and 18.07%.

(3) XRD, SEM, and SEM-EDS analyses showed that the content of the 5-1-7 strength phase did not decrease with the addition of 50% RM into BMSC. However, the M-S-H gel was generated due to the reaction of active SiO_2_ with α-MgO, and the RM that did not participate in the reaction could fill the pore structure of BMSC and its microstructure became denser. Simultaneously, RM reduces the amount of unreacted MgO and Mg(OH)_2_ production in the BMSC and improves the strength.

(4) In this study, only five proportions of RM addition are considered. Whether more RM can be added in BMSC requires further research. Additionally, our study only focused on the mechanical properties, pore structure, and microstructure of BMSCs. Further research will focus on the durability, life cycle, environmental impact, and economy of BMSC with the addition of different proportions of red mud.

## Figures and Tables

**Table 1:** Mix proportions of BMSC.

Specimen	MgO (g)	Mud (g)	MgSO_4_·7H_2_O Solution (g)	C_6_H_8_O_7_ (g)
R0	1000	0	907.44	6
R10	1000	100	907.44	6
R20	1000	200	907.44	6
R30	1000	300	907.44	6
R40	1000	400	907.44	6
R50	1000	500	907.44	6

**Table 2:** Oxide composition of raw materials.

Raw Material	Mass Fraction of the Samples (%)								
	MgO	Al_2_O_3_	SiO_2_	P_2_O_5_	SO_3_	K_2_O	CaO	MnO	Fe_2_O_3_
Magnesia	93.8	0.36	3.99	0.03	0.09	0.02	1.47	0.02	0.29
Red mud	0.58	4.31	32.61	0.22	0.02	0.78	0.64	0.12	60.45

**Table 3:** Pore structure characteristics of the BMSC specimens cured for 28 days.

Specimen	Porosity (%)	Total Pore Volume (mL/g)	Average Pore Size (nm)	Total Pore Area (m^2^/g)	Apparent Density (g/mL)
R0	22.09	0.13	32.18	16.50	2.14
R50	18.97	0.10	31.92	12.90	2.28

## Data Availability

The original contributions presented in the study are included in the article, further inquiries can be directed to the corresponding author.
